# Single-Stage Microsurgical Clipping of Multiple Intracranial Aneurysms in a Patient with Cerebral Atherosclerosis: A Case Report and Review of Surgical Management

**DOI:** 10.3390/jcm14010269

**Published:** 2025-01-05

**Authors:** Corneliu Toader, Matei Serban, Razvan-Adrian Covache-Busuioc, Mugurel Petrinel Radoi, Ghaith Saleh Radi Aljboor, Horia Petre Costin, Milena-Monica Ilie, Andrei Adrian Popa, Radu Mircea Gorgan

**Affiliations:** 1 Department of Neurosurgery, “Carol Davila” University of Medicine and Pharmacy, 050474 Bucharest, Romania; corneliu.toader@umfcd.ro (C.T.); razvan-adrian.covache-busuioc0720@stud.umfcd.ro (R.-A.C.-B.); petrinel.radoi@umfcd.ro (M.P.R.); ghaith-saleh-radi.aljboor@rez.umfcd.ro (G.S.R.A.); horia-petre.costin0720@stud.umfcd.ro (H.P.C.); milena-monica.ilie0720@stud.umfcd.ro (M.-M.I.); andreiadrianpopa@stud.umfcd.ro (A.A.P.); radu.gorgan@umfcd.ro (R.M.G.); 2 Department of Vascular Neurosurgery, National Institute of Neurology and Neurovascular Diseases, 077160 Bucharest, Romania; 3 Puls Med Association, 051885 Bucharest, Romania; 4 Department of Neurosurgery, Clinical Emergency Hospital “Bagdasar-Arseni”, 041915 Bucharest, Romania

**Keywords:** multiple intracranial aneurysms, microsurgical clipping, cerebral atherosclerosis, single-stage surgery, aneurysm management, middle cerebral artery aneurysm, pericallosal artery aneurysm, postoperative ischemia prevention

## Abstract

The management of multiple intracranial aneurysms presents significant clinical challenges, particularly when complicated by underlying conditions such as cerebral atherosclerosis. This case report highlights the successful treatment of a 66-year-old female diagnosed with three intracranial aneurysms located in the right middle cerebral artery (MCA), pericallosal artery, and M2 segment. The patient also had a history of systemic atherosclerosis and right-sided breast cancer, factors that increased the complexity of surgical intervention. The aim of this report is to demonstrate the efficacy of single-stage microsurgical clipping in managing multiple aneurysms with favorable outcomes in a complex patient profile. **Methods**: The patient underwent right-sided pterional craniotomy for microsurgical clipping of all three aneurysms during a single-stage procedure. Two aneurysms in the MCA were clipped using Yasargil clips, and a third aneurysm located at the bifurcation of the pericallosal artery was also secured with a clip. The procedure was performed under microscopic visualization, with meticulous dissection of the atherosclerotic vessels and careful intraoperative hemostasis. Postoperative care involved proactive perioperative management, including blood pressure control and vigilant neurological monitoring. **Results**: Postoperative imaging at three months confirmed proper clip placement with no evidence of residual aneurysm filling or ischemic complications. The patient exhibited a full neurological recovery, with no deficits or further complications, highlighting the effectiveness of the surgical approach in managing multiple aneurysms concurrently. **Conclusions**: This case supports the use of single-stage microsurgical clipping as an effective treatment for patients with multiple intracranial aneurysms, even in the presence of complicating factors such as atherosclerosis. A meticulous surgical technique and perioperative management are critical to achieving favorable outcomes and reducing the risk of delayed ischemia or other postoperative complications.

## 1. Introduction

Intracranial aneurysms represent a potentially life-threatening condition due to the risk of rupture, which can lead to subarachnoid hemorrhage—a complication associated with high morbidity and mortality. It is estimated that the prevalence of unruptured intracranial aneurysms (UIAs) in the general population is approximately 3–5%, with many cases remaining asymptomatic until rupture occurs. The risk of rupture, although variable, is influenced by several factors, including aneurysm size, location, and morphology and underlying vascular conditions such as cerebral atherosclerosis [1].

Aneurysms located at arterial bifurcations, such as the middle cerebral artery (MCA) and the pericallosal artery, are particularly prone to rupture due to increased hemodynamic stress. Recent studies have demonstrated that the MCA bifurcation is one of the most common sites for aneurysm formation, with the risk of rupture strongly correlating with aneurysm size and morphology [2]. Furthermore, the presence of cerebral atherosclerosis, which results in vascular wall weakening and increased hemodynamic burden, has been implicated in both the progression and rupture of intracranial aneurysms [3]. Atherosclerotic changes in cerebral vessels may also complicate the surgical management of these aneurysms, leading to increased technical difficulty and a heightened risk of postoperative complications [4].

Advances in microsurgical and endovascular techniques have significantly improved both the management and prognosis of patients with intracranial aneurysms. Microsurgical clipping remains the gold standard for treating aneurysms in accessible locations, particularly within the anterior circulation, where clipping has been shown to provide durable aneurysm exclusion with low recurrence rates. However, the surgical management of multiple aneurysms in a single patient presents unique challenges, especially when they are located in different vascular territories, as demonstrated in the case presented here [5].

The presence of multiple intracranial aneurysms is reported in approximately 15–30% of patients with aneurysmal disease, and their management requires a highly tailored approach. Surgical intervention, particularly in patients with a complex vascular history, demands a precise technique and thorough planning to ensure the successful exclusion of all aneurysms while minimizing risks to adjacent vascular structures [6]. Moreover, the increasing prevalence of atherosclerosis, driven by an aging population and comorbid conditions such as hypertension and hyperlipidemia, further complicates both the natural history and treatment of intracranial aneurysms [7].

In this case report, we describe the successful management of a 66-year-old female diagnosed with multiple right-sided intracranial aneurysms—two located along the right middle cerebral artery and one at the bifurcation of the right pericallosal artery. The patient’s cerebral and systemic atherosclerosis, combined with her history of right-sided breast cancer, underscores the complexity of this case. We discuss the surgical challenges, particularly in relation to the precise clipping of aneurysms in high-risk locations, and review the postoperative outcomes following successful intervention. This report contributes to the growing body of literature on the management of multiple intracranial aneurysms in patients with comorbid atherosclerotic disease.

## 2. Case Presentation

A 66-year-old female presented with an incidental diagnosis of multiple intracranial aneurysms following routine cerebral imaging. The patient was asymptomatic at the time of diagnosis, with no complaints of headaches, neurological deficits, or history of vascular events such as subarachnoid hemorrhage. Her medical history was significant for cerebral and systemic atherosclerosis and a prior diagnosis of right-sided breast cancer, which had been surgically treated. There was no family history of cerebrovascular disease or aneurysms.

**Figure 1:**
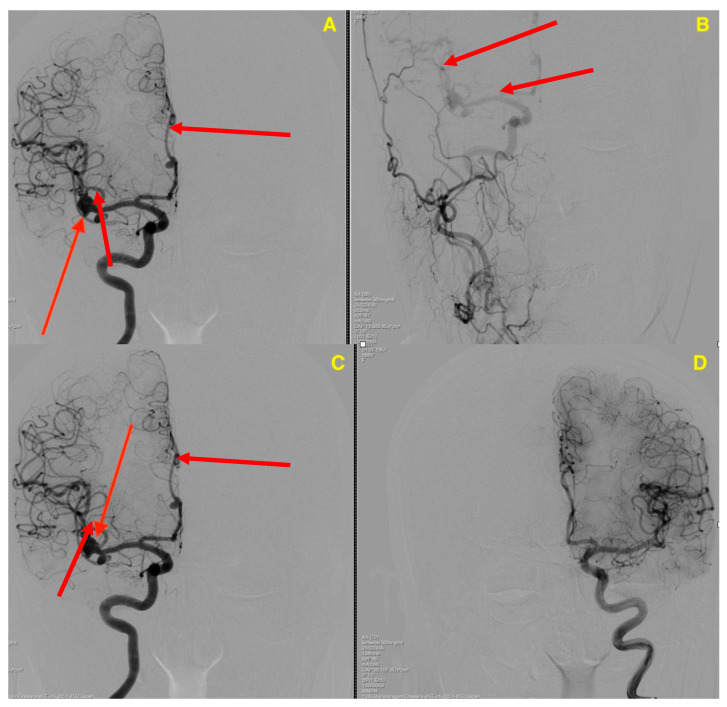
Preoperative cerebral angiography. (**A**,**C**) Right middle cerebral artery (MCA) aneurysm marked by red arrows, located at the MCA bifurcation. (**B**) Aneurysm in the right M2 segment. (**D**) Aneurysm located in the right pericallosal artery.

Preoperative cerebral angiography (Figure 1) revealed three intracranial aneurysms, all located in the right hemisphere. These included a large aneurysm at the right MCA bifurcation, a second aneurysm along the right M2 segment, and a third aneurysm in the right pericallosal artery, a branch of the anterior cerebral artery (ACA).

**Figure 2:**
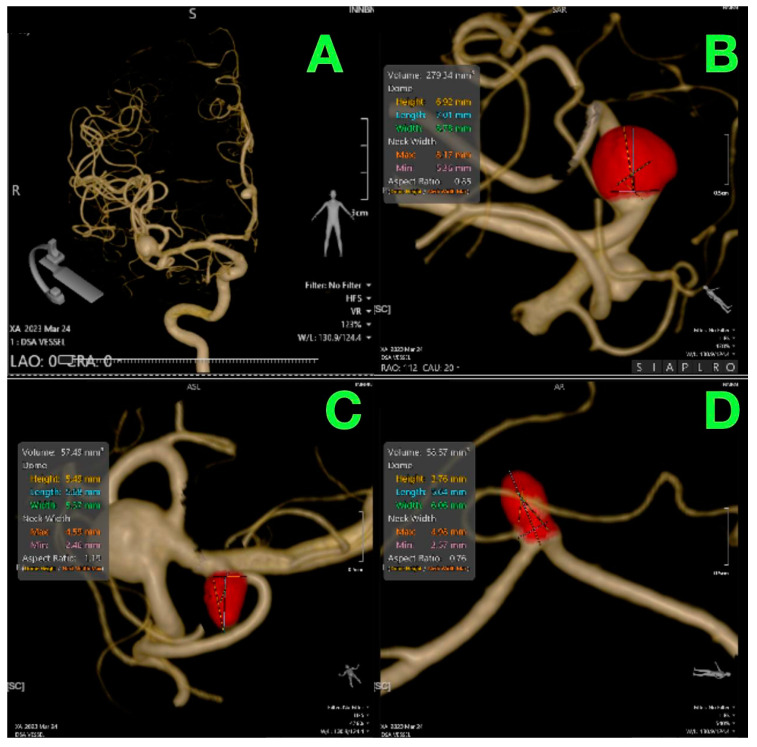
Preoperative 3D angiography reconstructions: (**A**) 3D vascular reconstruction showing the arterial tree of the right hemisphere; (**B**) right MCA bifurcation aneurysm, with a volume of 279.34 mm^3^, height of 6.92 mm, and neck width of 3.17 mm; (**C**) right M2 segment aneurysm, with a volume of 55.43 mm^3^, height of 5.34 mm, and neck width of 4.51 mm; (**D**) right pericallosal aneurysm, with a volume of 55.57 mm^3^, height of 3.76 mm, and neck width of 4.06 mm.

The largest aneurysm, located at the bifurcation of the right MCA, was clearly visualized in angiography reconstructions (Figure 2), specifically in panel B. The aneurysm, with a measured volume of 279.34 mm^3^, posed a significant rupture risk due to its size and location. The MCA supplies a large portion of the lateral cerebral hemisphere, including regions critical for motor and sensory function, particularly involving the contralateral upper extremities and face. Although the patient was asymptomatic, the aneurysm’s location near vital cortical structures raised concern for future complications, such as motor deficits, sensory loss, or aphasia if rupture were to occur. The underlying cerebral atherosclerosis further compounded the risk, as it can contribute to vascular wall weakness and increased aneurysmal instability.

The second aneurysm, found in the right M2 segment of the MCA (Figure 2, panel C), measured 55.43 mm^3^. While smaller in size, aneurysms in this location can still lead to significant neurological consequences if ruptured, given the M2 segment’s involvement in supplying deeper cortical areas, including parts of the temporal and insular cortices. These areas are responsible for higher cognitive functions, sensory integration, and motor control. The presence of systemic atherosclerosis could also increase the likelihood of aneurysmal growth or rupture in this region.

The third aneurysm, located in the right pericallosal artery (Figure 2, panel D), was measured at 55.57 mm^3^. This artery supplies the medial aspects of the frontal and parietal lobes, including the areas responsible for motor control of the lower extremities and aspects of executive functioning. Rupture of an aneurysm in this region could lead to contralateral lower extremity weakness or paralysis, along with potential cognitive or behavioral changes due to involvement of the frontal lobe. Given the patient’s history of cerebral atherosclerosis, the risk of rupture, even in smaller aneurysms, was a significant concern.

The patient’s neurological examination was unremarkable. She exhibited no motor or sensory deficits, and her reflexes, coordination, and gait were all within normal limits. Cranial nerve examination revealed no abnormalities, and there were no meningeal signs. Her cognitive functions were intact, with the patient fully oriented to time, place, and self. Despite her asymptomatic status, the presence of three aneurysms, combined with her history of cerebral and systemic atherosclerosis, warranted careful monitoring and a thorough discussion of treatment options.

Given the high risk of rupture associated with the right MCA bifurcation aneurysm, a multidisciplinary team was consulted to evaluate treatment options. Surgical intervention, such as microsurgical clipping, or endovascular options, such as coiling, were considered based on the aneurysm size, morphology, and anatomical complexity. The patient’s underlying atherosclerosis was also factored into the decision-making process, as it could affect both the growth of the aneurysms and the risk of intraoperative or postoperative complications. Long-term follow-up with regular imaging was planned to monitor the aneurysms for any changes in size or stability.

A right-sided pterional craniotomy was performed to address the complex vascular pathology involving multiple aneurysms in the right anterior circulation. This approach, though standard for accessing the anterior and middle cerebral arteries, demands surgical finesse, particularly in cases involving multiple aneurysms with varied locations and sizes. The craniotomy was executed with precision, providing optimal exposure while minimizing brain retraction. The dura mater was elevated and suspended circumferentially, and then incised using a basal pedicle to provide swift yet controlled access to the underlying vessels.

Under the operative microscope, the right Sylvian cistern was entered, and immediate attention was directed to the first and most clinically significant aneurysm at the bifurcation of the right MCA. The aneurysm measured approximately 7 mm in diameter, a size that significantly increases the risk of rupture under physiological stress. The location at the MCA bifurcation, a point of high-flow turbulence, added further complexity to the case. Given the delicate nature of this aneurysm, precise microsurgical dissection was performed to mobilize adjacent vessels, preserving the integrity of critical perforators. A Yasargil 7 mm clip was expertly placed across the aneurysmal neck, ensuring complete exclusion from the circulation while maintaining the flow through the parent artery.

Moving distally, the second aneurysm was identified along the distal right M2 segment, measuring 2.7 mm in diameter. Though smaller, its distal location presented a unique challenge due to the narrow operative corridor and the fine caliber of the involved vessels. Careful dissection along the Sylvian fissure allowed for safe exposure of the aneurysm without disturbing the surrounding neural structures. A Yasargil 5 mm clip was delicately placed on the aneurysmal neck, securing the aneurysm while preserving the integrity of the distal vasculature.

The procedure then progressed to the right pericallosal cistern, where the third aneurysm was located at the bifurcation of the right pericallosal artery. This aneurysm, measuring 4 mm, posed a distinct technical challenge due to its proximity to the corpus callosum and the deep location within the interhemispheric fissure. This region, with its smaller caliber arteries and close relationship to critical cortical areas, demands the utmost surgical precision. A Yasargil 3 mm clip was applied to the aneurysmal neck with careful attention to avoid compromising the adjacent vasculature or inducing retraction injury to the surrounding parenchyma.

**Figure 3:**
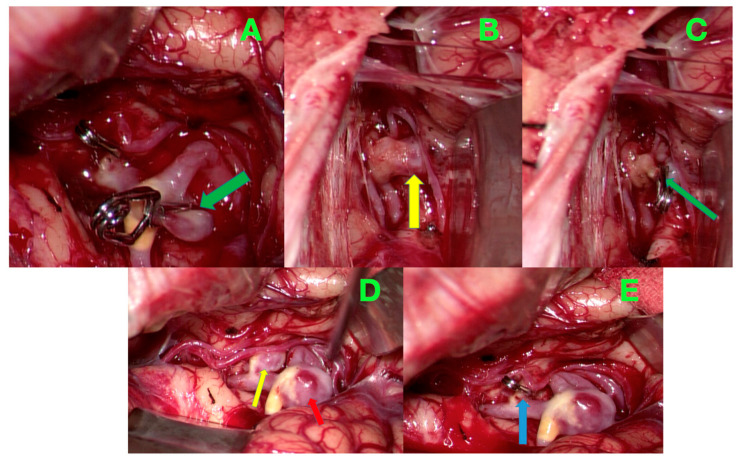
The intraoperative images demonstrate the step-by-step microsurgical management of intracranial aneurysms in a patient with cerebral atherosclerosis. (**A**) Initial visualization of the aneurysms located at the MCA bifurcation and along the M2 segment, highlighting the exposure of the vascular anatomy. The green arrow indicates the aneurysm neck at the MCA bifurcation. (**B**) Clipping of the aneurysm along the M2 segment, with meticulous preservation of the emerging vascular branches to maintain distal perfusion and vessel patency. The yellow arrow points to the applied clip securing the M2 segment aneurysm. (**C**) Clipping of the MCA bifurcation aneurysm, emphasizing the preservation of bifurcation anatomy and normal blood flow through the MCA branches. The green arrow shows the clipped aneurysm at the MCA bifurcation. (**D**) Identification of the right pericallosal artery aneurysm at the emergence of the callosal marginal artery, demonstrating the challenges posed by its deep-seated location. The red arrow highlights the aneurysm neck, and the yellow arrow points to the parent vessel. (**E**) Successful clipping of the pericallosal artery aneurysm, with precise attention to preserving the integrity and blood flow of the callosal marginal artery. The blue arrow marks the applied clip on the pericallosal aneurysm.

Throughout the procedure, meticulous hemostasis was maintained using electrocautery, Surgicel, and tamponade to ensure a clear operative field. Every effort was made to limit brain retraction and avoid unnecessary manipulation of the surrounding structures. Once all three aneurysms were successfully secured, the bone flap was meticulously repositioned and anchored. Closure proceeded in anatomical layers, maintaining the integrity of both the dura and scalp to reduce the risk of postoperative complications. A sterile dressing was applied, concluding the procedure. The intraoperative images presented in Figure 3 highlight critical steps in the microsurgical clipping of the three intracranial aneurysms.

The complexity of this case was driven by the multiple aneurysms in varied locations, each presenting unique challenges in terms of exposure, clipping, and avoidance of adjacent critical structures. The ability to perform safe and effective clipping in this context underscores the importance of surgical expertise, detailed anatomical knowledge, and the use of advanced microsurgical techniques. The procedure was completed without complications, and all aneurysms were successfully excluded from the circulation, minimizing the patient’s risk of future rupture.

At the three-month postoperative follow-up, the patient exhibited an excellent clinical recovery. She remained neurologically intact, with no reports of headaches, neurological deficits, or other concerning symptoms. A non-contrast CT scan of the head was performed to evaluate the positioning of the aneurysm clips and assess for any postoperative complications.

**Figure 4:**
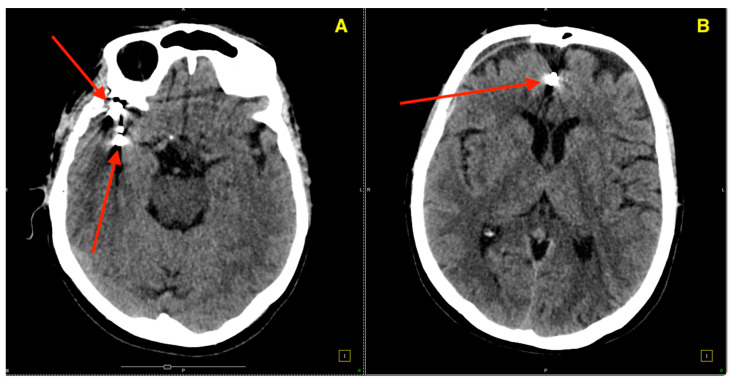
Three-month postoperative non-contrast CT scan. (**A**) Postoperative CT showing the aneurysm clips at the middle cerebral artery bifurcation (upper arrow) and the distal M2 segment (lower arrow), demonstrating proper placement without evidence of migration or residual filling. (**B**) Postoperative CT highlighting the aneurysm clip at the pericallosal artery, showing accurate positioning with no surrounding ischemic changes.

The CT scan (Figure 4) demonstrated optimal placement of all three aneurysm clips. Panel A shows the clips applied to the right MCA, clearly visible without evidence of migration or impingement on the surrounding vessels. The clips at the bifurcation and distal M2 segment were in ideal positions, securing the aneurysms while preserving the patency of the MCA. Panel B illustrates the clip placed on the right pericallosal artery aneurysm, also in an optimal position with no evidence of residual aneurysmal filling or vascular compromise. Importantly, there were no signs of ischemia, hemorrhage, or any other complications related to the surgery.

The patient’s smooth recovery and the stable radiographic findings confirm the effectiveness of the surgical intervention. The successful exclusion of the aneurysms from the cerebral circulation has markedly reduced the patient’s risk of future rupture, ensuring a favorable prognosis.

To provide a comprehensive understanding of the patient’s recovery and quality of life, we conducted assessments during the three-month postoperative follow-up. The patient reported full functional independence, with no neurological deficits, cognitive impairments, or limitations in daily activities. Her quality of life remained high, and she expressed satisfaction with the outcomes of the procedure. Follow-up visits included neurological evaluations and assessments of any cardiovascular symptoms, given her history of cerebral and systemic atherosclerosis. Postoperative imaging confirmed stable clip placement, with no evidence of residual aneurysm filling, migration, or ischemic complications. No further complications, such as delayed ischemia or rebleeding, were noted during this period, indicating a stable short-term postoperative course. These outcomes reflect the efficacy of single-stage microsurgical clipping and underscore the importance of precise surgical techniques and vigilant perioperative care in optimizing patient recovery and early prognosis.

In view of her atherosclerotic condition, the patient was advised to continue with regular neurological monitoring and cardiovascular risk management to mitigate the potential for late-onset vascular complications. This case underscores the importance of a tailored, multidisciplinary approach in managing complex aneurysm cases and highlights the potential for favorable long-term outcomes with appropriate surgical and postoperative care. The findings reinforce that single-stage microsurgical clipping can be a durable and effective solution, significantly reducing the risk of aneurysm recurrence and promoting a high quality of life following surgery.

In summary, this case illustrates that, while single-stage microsurgical clipping offers a robust solution for multiple aneurysms, particularly in complex patients, careful management of potential complications—both intraoperatively and postoperatively—is vital.

## 3. Discussions

Despite the favorable outcomes observed in this case, it is essential to recognize and address the potential complications associated with single-stage microsurgical clipping, especially in patients with cerebral atherosclerosis. One of the primary risks in such cases is delayed cerebral ischemia, a complication often exacerbated by atherosclerotic vessel changes that can impair blood flow and increase susceptibility to ischemic events following surgery. To mitigate this risk, careful intraoperative handling of fragile, atherosclerotic vessels is critical, as is the meticulous placement of clips to avoid compromising adjacent arteries.

Additionally, postoperative complications such as vasospasm and thromboembolic events remain a concern, given the patient’s underlying vascular condition. Proactive perioperative management, incorporating strict blood pressure regulation and, when suitable, the use of antiplatelet therapy, was crucial in averting these complications in the current case. Consistent follow-up with neurological assessments further enabled the early identification and treatment of any indications of vascular insufficiency. The risk of rebleeding or aneurysm recurrence, although low with microsurgical clipping, was another potential complication that influenced the decision for ongoing monitoring. The durable nature of clipping minimizes this risk compared to other interventions; however, patients with atherosclerosis may experience accelerated vascular degeneration, necessitating continued surveillance for late-onset complications.

The management of multiple intracranial aneurysms in the presence of cerebral atherosclerosis presents unique clinical and surgical challenges. In this case, the successful exclusion of three aneurysms—two involving the right MCA and one at the bifurcation of the right pericallosal artery—highlights both the complexity and technical precision required for effective treatment [8]. Although microsurgical clipping remains the gold standard for aneurysms, the presence of atherosclerotic changes significantly complicates both the surgical procedure and the long-term prognosis [9].

Cerebral atherosclerosis is widely recognized as a critical factor in the development, growth, and rupture of intracranial aneurysms. Atherosclerosis weakens arterial walls through mechanisms such as intimal thickening, endothelial dysfunction, and calcification, increasing the risk of aneurysm formation and rupture [10]. Several studies have confirmed that atherosclerotic aneurysms are more prone to rupture and are more challenging to manage surgically, particularly due to vessel fragility and reduced elasticity [11,12]. Studies demonstrated that patients with atherosclerosis are at a significantly higher risk of aneurysm rupture, emphasizing the need for a tailored surgical approach in this population [10,13].

Atherosclerosis fundamentally reshapes the pathophysiology and management of intracranial aneurysms, creating a dynamic interplay of structural fragility and surgical complexity. Beyond the well-established processes of endothelial dysfunction and arterial calcification, emerging evidence highlights how atherosclerosis perpetuates chronic inflammatory states that destabilize aneurysmal walls through enzymatic degradation and oxidative stress [14]. This pro-inflammatory environment significantly accelerates aneurysm growth and rupture risk, even in cases where aneurysms might otherwise be considered stable. The challenges posed by atherosclerosis are particularly pronounced in hemodynamically complex sites such as arterial bifurcations, where turbulent blood flow exacerbates wall shear stress and further compromises vascular integrity. Studies also point to impaired collateral circulation in atherosclerotic patients, which heightens susceptibility to ischemic events during surgical interventions [15].

In the present case, the combination of advanced atherosclerosis and multiple aneurysms in distinct, high-risk locations presented a unique challenge. Managing fragile, calcified vessels required precision dissection, careful clip placement, and the use of advanced intraoperative tools to maintain vessel patency. While staged surgeries are often favored to reduce procedural risks, the decision to proceed with a single-stage approach not only minimized cumulative anesthesia exposure but also eliminated the potential rupture risk of untreated aneurysms [12]. This case exemplifies the importance of tailoring surgical strategies to the individual patient’s pathology, using a combination of meticulous planning, intraoperative adaptability, and interdisciplinary expertise to address the compounded risks of atherosclerosis and multiple aneurysms.

The novelty of this case lies in the successful treatment of multiple aneurysms in a patient with both systemic and cerebral atherosclerosis. The first aneurysm, located at the bifurcation of the right MCA, presented a particularly challenging scenario due to the increased hemodynamic stress in this region. Arterial bifurcations are prone to aneurysm formation and rupture due to complex blood flow dynamics, which exacerbate wall shear stress [16]. The successful clipping of this aneurysm required careful microsurgical dissection to avoid damage to critical perforators and neighboring vascular structures [17].

In this case, the ‘squeeze play’ technique was pivotal in managing the complex aneurysms associated with atherosclerosis. This method, characterized by the gradual and controlled placement of the aneurysm clip while manipulating the vessel walls to achieve optimal closure, allowed for precise exclusion of the aneurysms without compromising the integrity of the adjacent vessels [18]. Particularly for the MCA bifurcation aneurysm, where atherosclerotic changes increased the risk of incomplete clipping or vessel injury, the ‘squeeze play’ approach provided the necessary control to ensure secure occlusion while preserving perforating branches. This technique has been documented as especially useful in cases with calcified or fragile vessel walls, where direct clipping can risk intimal damage or incomplete closure [19]. Its application in this case highlights the importance of incorporating advanced microsurgical techniques to navigate the challenges posed by atherosclerotic vessels and ensure favorable surgical outcomes.

Computational fluid dynamics studies have further highlighted that bifurcation aneurysms, like the one described here, are subject to turbulent flow patterns that contribute to their rupture risk and complicate their surgical management [20].

The second aneurysm, located in the distal M2 segment, although smaller, required precise microsurgical techniques due to its location in a narrow operative corridor with fragile, small-caliber vessels. Although smaller aneurysms are generally associated with a lower risk of rupture, the recent literature suggests that aneurysms under 3 mm, particularly in the presence of risk factors such as atherosclerosis, may still warrant aggressive surgical intervention [21]. The successful management of this aneurysm, despite its distal location and size, underscores the critical importance of technical expertise in microsurgery.

The third aneurysm, situated at the bifurcation of the right pericallosal artery, presented additional complexity due to its deep location within the interhemispheric fissure. Aneurysms in this region are rare and often associated with higher morbidity due to their proximity to critical cortical areas and the corpus callosum. Dissection within the pericallosal cistern requires a nuanced understanding of microanatomy, as the risk of injuring surrounding structures can lead to significant neurological deficits, including cognitive impairment or disconnection syndromes [22]. While there are limited data on the management of pericallosal artery aneurysms, the successful exclusion of this aneurysm without complications in this case highlights the importance of advanced microsurgical techniques.

Endovascular approaches, such as coiling and flow diversion, have revolutionized the treatment of intracranial aneurysms, particularly those located in deep or otherwise challenging locations. However, in this case, microsurgical clipping was selected due to the favorable accessibility of the aneurysms and the superior long-term durability of clipping. Several large-scale studies, including the ISAT trial, have shown that while endovascular coiling may be effective for many aneurysms, particularly those in the posterior circulation, clipping offers lower recurrence rates in aneurysms with wide necks or those located at bifurcation points [23]. Moreover, the current literature suggests that microsurgical clipping remains the preferred method in younger patients or those with long life expectancies, where the durability of the intervention is a key consideration [24].

The presence of multiple aneurysms, which occurs in approximately 15–30% of patients with aneurysmal disease, adds another layer of complexity to the management plan [25]. Some advocate for staged treatment, addressing aneurysms one at a time. However, in this case, all three aneurysms were treated during a single procedure, reducing the risks associated with multiple surgical interventions and general anesthesia exposures. This approach minimized the potential for rupture of untreated aneurysms during the follow-up and ensured a more immediate resolution of the patient’s aneurysmal burden. Performing a one-stage surgery, though demanding, provided a definitive solution for the patient’s vascular pathology and demonstrated the feasibility of such an approach in experienced hands.

The patient’s underlying cerebral and systemic atherosclerosis posed additional risks both during and after surgery. Not only is atherosclerosis a risk factor for aneurysm formation, but it also increases the likelihood of postoperative complications, such as vasospasm, delayed ischemia, and rebleeding. A study by Juvela et al. highlighted that patients with atherosclerosis are more prone to delayed cerebral ischemia after aneurysm surgery, reinforcing the importance of vigilant postoperative monitoring and management [26]. In this case, meticulous intraoperative handling of the atherosclerotic vessels, combined with proactive perioperative management, ensured a favorable outcome with no postoperative ischemic complications.

Recent studies have further underscored the advantages of single-stage microsurgical clipping over staged procedures for patients with multiple aneurysms, particularly when combined with complicating factors such as atherosclerosis [27,28]. The decision between single-stage and multi-stage microsurgical clipping of multiple intracranial aneurysms is multifaceted, requiring a nuanced evaluation of patient-specific factors and surgical complexities. Single-stage clipping offers several advantages, including the ability to address all aneurysms in a single operation, thereby reducing the cumulative exposure to anesthesia, shortening overall hospitalization, and eliminating the risk of rupture for untreated aneurysms during the interim period [25,29]. This approach is particularly advantageous for anatomically accessible aneurysms or patients whose comorbid conditions, such as atherosclerosis, make multiple procedures particularly risky. However, the challenges of single-stage clipping include prolonged operative times, which can heighten the risk of brain swelling, ischemia, and fatigue-related complications for the surgical team [30,31]. Advances in intraoperative imaging and monitoring, such as real-time Doppler and fluorescence angiography, have improved the safety profile of single-stage procedures, allowing for precise clip placement and continuous assessment of vascular patency [32].

Multi-stage clipping, in contrast, distributes the surgical burden across multiple sessions, reducing intraoperative stress and allowing for a focused approach to each aneurysm. This method is particularly beneficial when aneurysms are located in different vascular territories or involve high degrees of calcification or fragility [33,34]. However, the risks associated with staged interventions include the potential for the rupture of untreated aneurysms during the interval period, as well as the cumulative effects of repeated anesthesia and hospitalization [35]. These factors must be carefully weighed, especially in patients with advanced atherosclerosis, where vessel fragility can exacerbate procedural risks [36].

While staged surgeries may offer a stepwise approach that distributes procedural stress, they inherently expose patients to multiple anesthesia sessions, cumulative surgical risks, and the possibility of aneurysm rupture between interventions [37,38]. Single-stage clipping, when feasible, provides an immediate and comprehensive solution, significantly reducing the aneurysmal burden in one operation and minimizing the potential for interim complications [39,40].

In addition, emerging evidence suggests that single-stage approaches are associated with favorable long-term outcomes in cases with complex aneurysmal profiles, where precise control and direct vessel handling are essential [41,42,43]. A growing body of literature indicates that, in the context of atherosclerotic vessels and wide-necked aneurysms, single-stage clipping may offer a more definitive exclusion compared to staged or purely endovascular options [44,45,46].

In addition to the factors discussed, the decision to pursue microsurgical clipping over endovascular techniques was further influenced by the specific vascular pathology of the patient. The aneurysms presented with wide necks at bifurcation points, characteristics that pose challenges for flow diversion due to the risk of compromising adjacent branch arteries [47]. While flow diversion is effective for certain aneurysm types, particularly those in more distal locations or without critical branching vessels, the bifurcation sites involved in this case heightened the risk of incomplete exclusion or branch occlusion if treated with endovascular devices [48].

Furthermore, the patient’s atherosclerotic changes introduced additional complexities. Atherosclerosis can reduce vascular flexibility, which may hinder safe catheter navigation and device placement, thus increasing the risk of procedural complications. Microsurgical clipping, by contrast, allowed direct access to the aneurysms with precise control, particularly beneficial in handling atherosclerotic vessels where clip placement could be carefully managed to avoid intraoperative complications [49,50].

Finally, the multidisciplinary team prioritized durability and the need for a long-lasting solution, given the patient’s age and relatively high life expectancy. The choice of clipping reflects an evidence-based approach tailored to maximizing the patient’s long-term prognosis and minimizing the need for future interventions [51]. This case underscores the importance of individualized treatment planning, where anatomical and patient-specific factors guide the selection of optimal surgical strategies.

In reviewing the literature, various approaches to managing multiple intracranial aneurysms, particularly in cases involving three or more aneurysms, have demonstrated flexibility and effectiveness depending on patient-specific factors and aneurysm characteristics. Studies have shown that single-stage microsurgical clipping is frequently employed when the aneurysms are anatomically accessible and can be safely treated in a single operation (Table 1).

The patient’s history of right-sided breast cancer, while not directly related to her vascular condition, raises interesting considerations. Breast cancer and its associated treatments, particularly chemotherapy, have been linked to systemic inflammation and a prothrombotic state, both of which can exacerbate underlying vascular conditions such as atherosclerosis [59,60]. The successful management of this patient’s aneurysms, despite her oncological history, underscores the importance of a multidisciplinary approach to care, involving both vascular and oncological expertise.

## 4. Conclusions

In conclusion, this case illustrates the successful management of multiple intracranial aneurysms in a patient with significant vascular comorbidities, including cerebral and systemic atherosclerosis. The use of microsurgical clipping, despite the presence of vascular fragility, resulted in a favorable outcome with the secure exclusion of all aneurysms. This case highlights the ongoing relevance of microsurgical techniques in the management of complex aneurysms and emphasizes the need for individualized treatment strategies, particularly in patients with multiple comorbidities. Further research is warranted to explore long-term outcomes in patients with multiple aneurysms and to optimize treatment strategies in this challenging patient population.

## Figures and Tables

**Table 1:** Summary of case reports and studies on the management of multiple intracranial aneurysms. The table highlights key studies involving patients with more than two intracranial aneurysms treated through single-stage or multi-stage approaches. Details include the number of aneurysms, their anatomical locations (e.g., MCA: middle cerebral artery; ICA: internal carotid artery; PCoA: posterior communicating artery; ACoA: anterior communicating artery), treatment methods (e.g., microsurgical clipping, endovascular treatment, or hybrid approaches), clinical outcomes (e.g., full recovery or stable occlusion), complications (e.g., ischemia, vasospasm), and follow-up durations. This compilation underscores the diversity of treatment strategies and their associated results in managing complex aneurysmal cases.

Author(s) and Year	Number of Aneurysms	Locations	Treatment Approach	Outcome	Complications	Follow-Up Duration
Seo et al., 2022 [25]	5	MCA bifurcation, ACA, PCoA, ICA	Single-stage surgery with simultaneous craniotomies	Good recovery	None	12 months
Choque-Velasquez et al., 2017 [34]	7	MCA, ICA, ACA, PCoA	Single-stage surgery via one craniotomy	Full recovery	None	12 months
Kiran et al., 2020 [52]	3	MCA, ACoA, ICA	Single-stage microsurgical clipping	Stable occlusion	None	12 months
Choi et al., 2010 [53]	3	MCA bifurcation, ACA, PCoA	Microsurgical clipping	Full recovery	None	12 months
Juvela et al., 2021 [54]	3	MCA, ACoA, PCA	Staged surgeries with clipping	Delayed ischemia	None	18 months
Jeon et al., 2014 [55]	3	MCA bifurcation, PCA, ICA	Endovascular coiling	Stable occlusion	Mild vasospasm	6 months
Xu, 2020 [35]	3	ACA, ICA, PCoA	Hybrid approach: clipping and coiling	Full recovery	None	36 months
Ulmer et al., 2024 [56]	4	MCA, ACoA, ICA, ACA	Single-stage keyhole clipping	Stable occlusion	Minor transient ischemia	24 months
Andic et al., 2017 [57]	6	MCA, ACoA, ICA, PCoA, pericallosal	Single-stage endovascular treatment	Stable occlusion	None	18 months
Santiago et al., 2024 [58]	3	MCA, ICA, PCoA	Single-stage clipping via pterional approach	Full recovery	None	12 months

## Data Availability

The data presented in this study are available on request from the corresponding author.
